# Ultraviolet Radiation-Induced Mitochondrial Disturbances Are Attenuated by Metabolites of Melatonin in Human Epidermal Keratinocytes

**DOI:** 10.3390/metabo13070861

**Published:** 2023-07-20

**Authors:** Chantal E. Holtkamp, Dawid Warmus, Klaudia Bonowicz, Maciej Gagat, Kinga Linowiecka, Agnieszka Wolnicka-Glubisz, Russel J. Reiter, Markus Böhm, Andrzej T. Slominski, Kerstin Steinbrink, Konrad Kleszczyński

**Affiliations:** 1 Department of Dermatology, University of Münster, Von-Esmarch-Str. 58, 48149 Münster, Germany; chantal.holtkamp@uni-muenster.de (C.E.H.); boehmma@ukmuenster.de (M.B.); kerstin.steinbrink@ukmuenster.de (K.S.); 2 Department of Biophysics and Cancer Biology, Faculty of Biochemistry, Biophysics and Biotechnology, Jagiellonian University, Gronostajowa 7, 30-387 Krakow, Poland; dawid.warmus@unibe.ch (D.W.); a.wolnicka-glubisz@uj.edu.pl (A.W.-G.); 3 Department of Histology and Embryology, Collegium Medicum in Bydgoszcz, Nicolaus Copernicus University in Torun, 85-092 Bydgoszcz, Poland; klaudia.bonowicz@cm.umk.pl (K.B.); mgagat@cm.umk.pl (M.G.); 4 Department of Human Biology, Faculty of Biological and Veterinary Sciences, Nicolaus Copernicus University, Lwowska 1, 87-100 Toruń, Poland; klinowiecka@umk.pl or kxl1082@med.miami.edu; 5 Phillip Frost Department of Dermatology & Cutaneous Surgery, University of Miami Miller School of Medicine, Miami, FL 33125, USA; 6 Department of Cell Systems and Anatomy, UT Health, Long School of Medicine, San Antonio, TX 78229, USA; reiter@uthscsa.edu; 7 Department of Dermatology, Comprehensive Cancer Center, University of Alabama at Birmingham, Birmingham, AL 35294, USA; aslominski@uabmc.edu; 8 Pathology and Laboratory Medicine Service, VA Medical Center, Birmingham, AL 35294, USA

**Keywords:** melatonin, kynurenic metabolites, indolic metabolites, ultraviolet radiation, human epidermal keratinocytes, mitochondria, oxidative stress, caspases, apoptosis

## Abstract

Melatonin (*N*-acetyl-5-methoxytryptamine) is recognized as an effective antioxidant produced by the pineal gland, brain and peripheral organs, which also has anti-inflammatory, immunomodulatory, and anti-tumour capacities. Melatonin has been reported as a substance that counteracts ultraviolet radiation B (UVB)-induced intracellular disturbances. Nevertheless, the mechanistic actions of related molecules including its kynurenic derivatives (*N*^1^-acetyl-*N*^2^-formyl-5-methoxykynurenine (AFMK)), its indolic derivatives (6-hydroxymelatonin (6(OH)MEL) and 5-methoxytryptamine (5-MT)) and its precursor *N*-acetylserotonin (NAS) are only poorly understood. Herein, we treated human epidermal keratinocytes with UVB and assessed the protective effect of the studied substances in terms of the maintenance of mitochondrial function or their radical scavenging capacity. Our results show that UVB caused the significant elevation of catalase (CAT) and superoxide dismutase (Mn-SOD), the dissipation of mitochondrial transmembrane potential (mtΔΨ), a reduction in ATP synthesis, and the enhanced release of cytochrome *c* into cytosol, leading subsequently to UVB-mediated activation of the caspases and apoptosis (appearance of sub-G_1_ population). Our findings, combined with data reported so far, indicate the counteracting and beneficial actions of melatonin and its molecular derivatives against these deleterious changes within mitochondria. Therefore, they define a path to the development of novel strategies delaying mitochondrial aging and promoting the well-being of human skin.

## 1. Introduction

Melatonin (*N*-acetyl-5-methoxytryptamine, MEL) is synthesized by pinealocytes and retinal photoreceptors in a cyclic pattern, with the highest levels occurring at night when these fluctuations play a key role in circadian rhythm regulation [1,2]. Melatonin and its metabolites are widely detected in almost all biological systems including animals, plants, microbes [3,4,5,6] and even in honey [7,8]. Melatonin is widely described as a neuroendocrine mediator with pleiotropic bioactivities such as neurotransmitter, hormonal, immunomodulator and biological modifier actions [9,10]. In clinical studies, melatonin was also shown to suppress ultraviolet radiation (UVR)-induced erythema of skin [11,12]. It exhibits UVR-protective actions through the scavenging or reduction of reactive oxygen species (ROS), especially quenching hydroxyl radicals [13,14,15,16]; additionally, it influences immune functions [17] and anti-inflammatory and anti-apoptotic activities [18,19]. These effects may or may not be mediated by cell-membrane-bound melatonin receptors (MT1 or MT2) which belong to the G-protein-coupled receptor superfamily found to form heterodimers [20]. Melatonin removes ROS by means of direct scavenging or converting them into less toxic products which include enzymes such as *γ*-glutamylcysteine synthetase (*γ*-GCS), heme oxygenase-1 (HO-1), and NADPH quinone dehydrogenase-1 (NQO1), but also glutathione peroxidase (GPx), catalase (CAT) and superoxide dismutase (Mn-SOD, Cu/Zn-SOD) [21,22,23,24]. 

High concentrations of melatonin are found in various tissues and cells, including the bone marrow [25], lymphocytes [26], retina [27], thymus [28], oocytes [29,30,31], follicular fluid [32], and skin [6,33,34]. Although it is differentially distributed in subcellular organelles due to its amphiphilic character [1,35], it is found in the highest concentration in the mitochondria [36,37]. This highly lipophilic molecule easily penetrates cellular membranes protecting proteins, enzymes, mitochondria, lipids and DNA against oxidative damage [22,38].

The importance of metabolites of melatonin has come into focus due to their ability to reduce the damage invoked by oxidative stress as well as their capability to stimulate antioxidant enzymes [39]. Although metabolites of melatonin can function as potent antioxidants [40], under some pathological conditions, their pro-oxidant activity was also reported [41]. The antioxidant actions are a result of their actions as free radical scavengers or inducers of anti-oxidative enzymes [2,39,42]. Indolic, kynurenic, and classical pathways are described as the main pathways of melatonin metabolism [12,42,43]; these pathways are activated in the skin under UVB exposure [44]. *N*-acetylserotonin (NAS) is produced from serotonin and serves as a melatonin precursor [6,45]. *N*^1^-acetyl-*N*^2^-formyl-5-methoxykynuramine (AFMK) is produced from melatonin via the kynurenic pathway [42,43], through interaction with H_2_O_2_ [39,46] or due to UVB exposure [39]. Melatonin metabolites also include 6-hydroxymelatonin (6(OH)MEL) and 5-methoxytryptamine (5-MT) produced via the indolic pathway [47].

Exposure of the skin to UVR, where UVB (290–320 nm) is absorbed into the epidermis and UVA (320–400 nm) is absorbed into the dermis, directly induces DNA damage [48]. Namely, exposure to UVR triggers the massive accumulation of cyclobutane pyrimidine dimers (CPDs) and to a lesser degree of 6-4-pyrimidine photoproducts (6-4-PPs) [1,13,14,15]. Moreover, UVR induces intracellular disturbances including changes in plasma membrane potential or the acidification of cytosol [18], inducing mitochondria-dependent apoptosis [49] and triggering anti-oxidative responses in cutaneous cells [31].

In this report, we investigated whether melatonin or its selected metabolites attenuate the deleterious effects of UVB on mitochondrial homeostasis in human epidermal keratinocytes. 

## 2. Materials and Methods

### 2.1. Reagents

Melatonin (MEL), its derivatives (5-MT, 6(OH)MEL, NAS), 1% penicillin-streptomycin solution (10,000 units of penicillin and 10 mg of streptomycin in 1 mL 0.9% NaCl), 3-(4,5-dimethylthiazol-2-yl)-2,5-diphenyltetrazolium bromide (MTT), 4′,6-diamidino-2-phenylindole (DAPI), acetone, bovine serum albumin (BSA), carbonyl cyanide-m-chlorophenylhydrazone (CCCP), dimethyl sulphoxide (DMSO), ethanol, HCl, isopropanol, methanol, NaCl, NP-40 lysis buffer, paraformaldehyde (PFA), RNase A, sodium dodecyl sulphate (SDS), sodium deoxycholate, and Tris-HCl (pH 7.6) were purchased from Sigma (St. Louis, MO, USA). AFMK and 0.05% trypsin/0.53 mM EDTA solution were provided by Cayman Chemical (Ann Arbor, MI, USA) and Thermo Fisher Scientific (Waltham, MA, USA), respectively. Keratinocyte growth medium 2 (KGM 2) was purchased from PromoCell (Heidelberg, Germany).

### 2.2. Cell Culture

Normal human epidermal keratinocytes (NHEK, passage 2, pooled donors) were supplied by PromoCell and cultured in KGM 2 medium supplemented with 1% penicillin–streptomycin solution in a humidified atmosphere of 5% CO_2_ at 37 °C prior to the desired assessments.

### 2.3. Pre-Incubation with Melatonin, Its Metabolites and UVB Exposure

Prior to the start of the experimental treatment, cells were seeded and cultured for 24 h. Thereafter, the culture medium was replaced with a medium containing MEL or its derivatives (AFMK, 5-MT, 6(OH)MEL, NAS). All substances were dissolved in absolute ethanol, diluted with phosphate-buffered saline (1 × PBS, pH 7.4) to yield 10^−2^ M stock solution, and further diluted in the culture medium to the final concentrations, i.e., 10^−3^, 10^−5^, 10^−7^, 10^−9^ M, for 1 h pre-incubation prior to the UVB exposure. Briefly, cells were washed twice with 1 × PBS to remove remnants of the culture medium, and 1 × PBS was added again before irradiation. NHEK were exposed to the UVB (range: 280–320 nm) using an irradiation bank consisting of six UV bulbs (TL12, Philips, The Netherlands). Cells were irradiated in a dose-dependent manner (25, 50, 75 mJ/cm^2^) compared to the sham-irradiated samples (0 mJ/cm^2^, control value), i.e., cells in a culture dish covered with aluminum foil as described previously [18,19,23]. After UVB exposure, cells were cultured and subjected to the following assessments.

### 2.4. Cell Viability Assay

Tested cells were seeded on 96-well plates (0.15 × 10^5^ cells/well in the culture medium) and grown to subconfluence (as judged from light microscopy); this was followed by a 1 h pre-incubation period with respective compounds and subsequent UVB exposure, and the MTT assay was performed using the previously described procedures [50]. MTT (5 mg/mL in 1 × PBS) was prepared in the culture medium (the final dilution, 1:10), 100 μL of assay reagent was added to each well, and cells were incubated for 3 h in a humidified atmosphere of 5% CO_2_ at 37 °C. The resultant formazan crystals were dissolved using 100 μL of isopropanol/0.04 N HCl, absorbance was measured at λ = 595 nm using the BioTek ELx808™ microplate reader (BioTek Instruments, Inc., Winooski, VT, USA), and the results were normalized to the control sample.

### 2.5. Crystal Violet Assessment

The proliferation of cutaneous cells was assessed using ready-to-use crystal violet (CV) solution (Cell Biolabs, Inc., San Diego, CA, USA). Cells were seeded on 12-well plates (0.1 × 10^6^ cells/well) and experiments were performed when the culture reached 70–80% confluence. At the desired time points, cells were washed twice with 1 × PBS, stained with 0.1% CV solution in 10% ethanol for 3 min at room temperature (RT), and remnants were washed again with 1 × PBS. Plates were photographed and quantified using the Image J 1.51f software (National Institute of Health, Bethesda, MD, USA). 

### 2.6. Evaluation of ATP Synthesis and Oxidative Stress-Related State 

Alterations in mitochondrial homeostasis and resultant oxidative stress were assessed using colorimetric assays for ATP synthesis, catalase (CAT) and superoxide dismutase (Mn-SOD) activity (BioVision, Inc., Milpitas, CA, USA). Briefly, cells were seeded on 6-well plates (0.3 × 10^6^ cells/well) and according to the specified incubation time points, cells were treated with the assay buffers included in the kits, and reaction was determined using the developer mix for 30 min (ATP assay), 10 min (CAT assay) at RT and for 20 min (Mn-SOD assay) at 37 °C. The absorbance was measured at 570 nm (ATP, CAT) and 450 nm (Mn-SOD) using the BioTek ELx808™ microplate reader. 

### 2.7. Evaluation of the Mitochondrial Transmembrane Potential (mtΔΨ) and Release of Cytochrome c into Cytosol

According to the specified time points, changes within the mtΔΨ were assessed using tetramethyl rhodamine methyl ester (TMRM) (Thermo Fisher Scientific). Briefly, cells were seeded on 6-well plates (0.3 × 10^6^ cells/well), grown to subconfluence, pre-incubated for 1 h with MEL or its metabolites, exposed to UVB, and cultured in a fresh culture medium. Thereafter, cells were labelled with TMRM (the final concentration being 10 nM) in a humidified atmosphere of 5% CO_2_ at 37 °C for 1 h. Fluorescence intensity was measured using the Cytek NL-3000 flow cytometer (Amsterdam, The Netherlands). The mean of TMRM fluorescence intensity was obtained from 20,000 cells using excitation at 552 nm and 578 nm emission settings. The obtained results were presented as percentages of the control values.

The visualization of the UVB-induced depolarization of mtΔΨ and mitochondrial cytochrome *c* release into cytosol was conducted using fluorescence microscopy as described earlier by Qin et al. [51]. Cells were placed onto glass cover slips and grown on 6-well plates. Cells were stained for 1 h with TMRM (the final concentration, 100 nM) in a humidified atmosphere of 5% CO_2_ at 37 °C, washed three times with pre-warmed 1 × PBS, and fixed for 15 min using 4% paraformaldehyde (PFA) at RT. Subsequently, cells were permeabilized with an ice-cold acetone/methanol (3:7) mixture for 20 min at −20 °C, and mounted in Fluoromount-G^®^ medium (SouthernBiotech, Birmingham, AL, USA). As the positive control for the depolarization of mtΔΨ, cells were treated for 10 min with 100 μM CCCP.

For cytochrome *c* release, followed by fixation and permeabilization, cells were washed three times with pre-warmed 1 × PBS, pre-incubated for 1 h with 1% BSA at RT, tagged with primary mouse monoclonal anti-cytochrome *c* Ab (1:100) (Abcam, Inc., Cambridge, MA, USA) and secondary goat anti-mouse polyclonal Ab conjugated with Alexa Fluor^®^ 488 (1:500) for 2 h at RT, and mounted in mounting medium. 

All fluorescent images were obtained using an inverted fluorescence microscope, Axio Observer Z1 (Zeiss, Göttingen, Germany) equipped with a Mercury Short-arc HXP 120 lamp, an Axiocam 503 mono camera, and with respective filters for the detection of TMRM (Exi: 550/25 nm; Emi: 605/70 nm) and cytochrome *c* (Exi: 470/40 nm; Emi: 525/50 nm).

### 2.8. Assessment of Activation of Caspases

Caspase 3/7 activity was measured as described earlier [52] using Caspase-Glo^®^ 3/7 Assay (Promega, Madison, WI, USA). Followed by experimental time points, cells were washed with 1 × PBS, scraped, lysed in 90 μL of RIPA buffer (25 mM Tris-HCl [pH 7.6], 150 mM NaCl, 1% NP-40, 1% sodium deoxycholate, 0.1% SDS), and stored at −20 °C. Next, 4 μg of protein adjusted to 40 μL with RIPA was mixed with 40 μL of Caspase-Glo^®^ 3/7 Reagent on a white-walled 96-well plate, thoroughly shaken for 1 min at 300 rpm, and kept in the dark for 90 min at RT. The resultant luminescence was measured using the Infinite M200 PRO microplate reader (Tecan, Männedorf, Switzerland).

### 2.9. Evaluation of Apoptotic Sub-G1 Population

Cells were seeded on 6-well plates (0.3 × 10^6^ cells/well) and treated as described above. Adherent and floating cells were pooled, washed twice with pre-warmed 1 × PBS, then fixed via vortexing in 1 mL of 70% ice-cold ethanol and maintained for 24 h at −20 °C. Cells were collected via centrifugation at 2000 r.p.m. for 5 min, and the cell pellet was stained with 1 μg/mL DAPI (1:2000 in 1 × PBS) and 0.5 mg/mL RNase A for 30 min at RT in the dark. The mean of DAPI fluorescence intensity was obtained using the Cytek NL-3000 flow cytometer from 20,000 cells using excitation at 358 nm and 461 nm emission settings, with gating out of doublets and clumps. The percentage of cells undergoing apoptosis was obtained from the distinct sub-G_1_ population.

### 2.10. Statistical Analysis

Data were expressed as pooled means + standard error of the mean (S.E.M.) of at least three independent experiments. Results were normalized and expressed as a percentage of the control value, i.e., sham-irradiated cells (0 mJ/cm^2^). Statistically significant differences between results were determined using univariate analysis of variance (ANOVA) or Student’s *t*-test and appropriate post hoc analysis using GraphPad Prism 7.05 software (La Jolla, CA, USA). A *p*-value of less than 0.05 was considered statistically significant.

## 3. Results

### 3.1. The Deleterious Effect of UVB on the Proliferation Ratio and Counteracting Action of Melatonin and Its Metabolites

**Figure 1:**
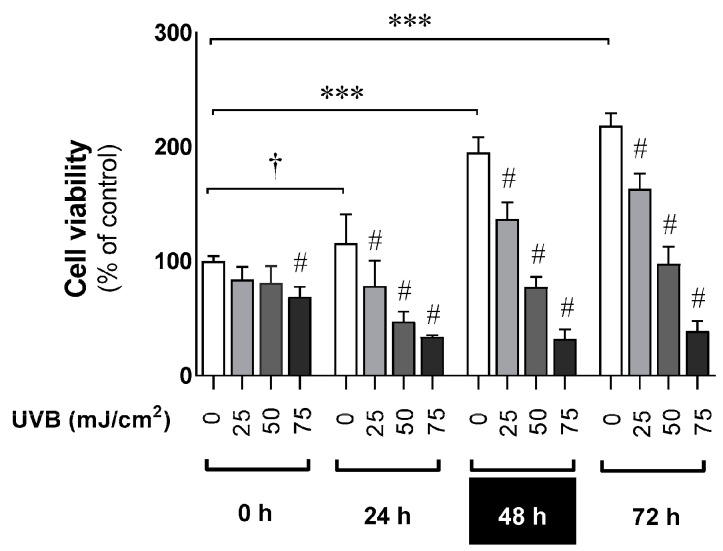
Assessment of cell viability of human epidermal keratinocytes exposed to UVB. Cells were irradiated in a dose-dependent manner and cultured post-UVB for up to 72 h. The MTT assay was employed as described in *Materials and Methods*. Data are presented as the mean +S.E.M. of four independent experiments. Values are normalized and expressed as a percentage of the control value—that is, a sham-irradiated sample (0 h 0 mJ/cm^2^). Statistically significant differences compared to control cells at 0 h are indicated as ^†^ *p* < 0.05, *** *p* < 0.001. Comparisons of respective UVB doses with sham-irradiated cells within subsequent time points (24, 48, 72 h) are indicated as significant with ^#^ *p* < 0.001. The obtained results determined the time point of 48 h (black rectangle) for further investigations of the counteracting effects of melatonin and its metabolites against UVB (25 mJ/cm^2^) presented in the figures below.

Human epidermal keratinocytes were exposed to UVB in a dose- and time-dependent manner (Figure 1). The obtained results revealed the significant decrease in cell viability compared to sham-irradiated cells. The reduction ranged from 17% to 59% (25 mJ/cm^2^), from 20% to 121% (50 mJ/cm^2^) and from 22% to 180% (75 mJ/cm^2^), while non-irradiated cells led to the distinct enhancement of cell growth by 15% (24 h), 95% (48 h) and 118% (72 h) compared to control cells at 0 h. 

**Figure 2:**
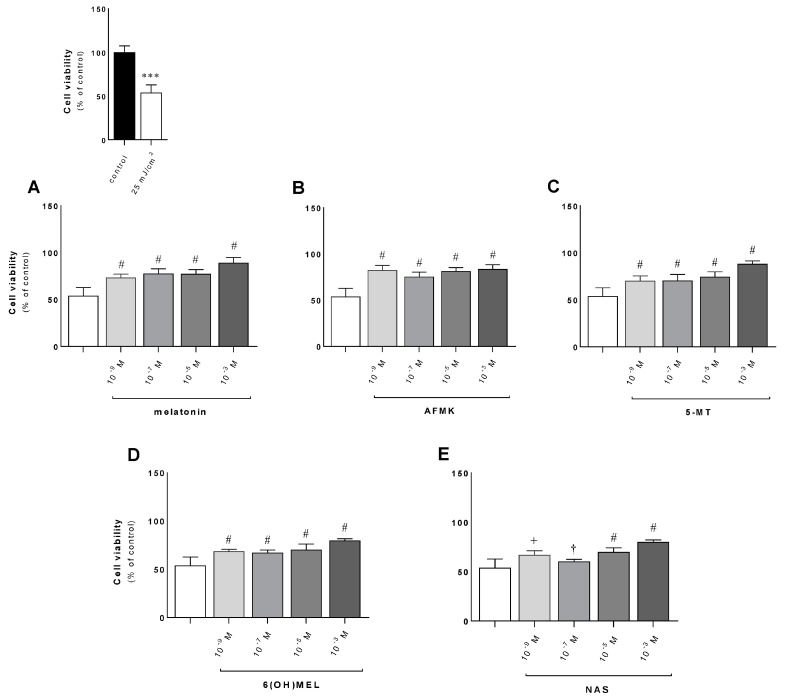
Evaluation of the protective effect of metabolites of melatonin against UVB exposure in human epidermal keratinocytes. Irradiated cells (25 mJ/cm^2^) were pre-incubated for 1 h with graded concentrations of the studied substances and assessed 48 h post-exposure for changes in cell viability as described in *Materials and Methods*. Data are presented as the mean +S.E.M. of four independent experiments (**A**–**E**). Statistically significant differences are indicated as *** *p* < 0.001 versus sham-irradiated cells (control sample) and as ^†^ *p*< 0.05, ^+^ *p* < 0.01, ^#^ *p* < 0.001 for subjected compounds versus UVB-exposed cells. Values are expressed as a percentage of sham-irradiated cells (0 h 0 mJ/cm^2^).

**Figure 3:**
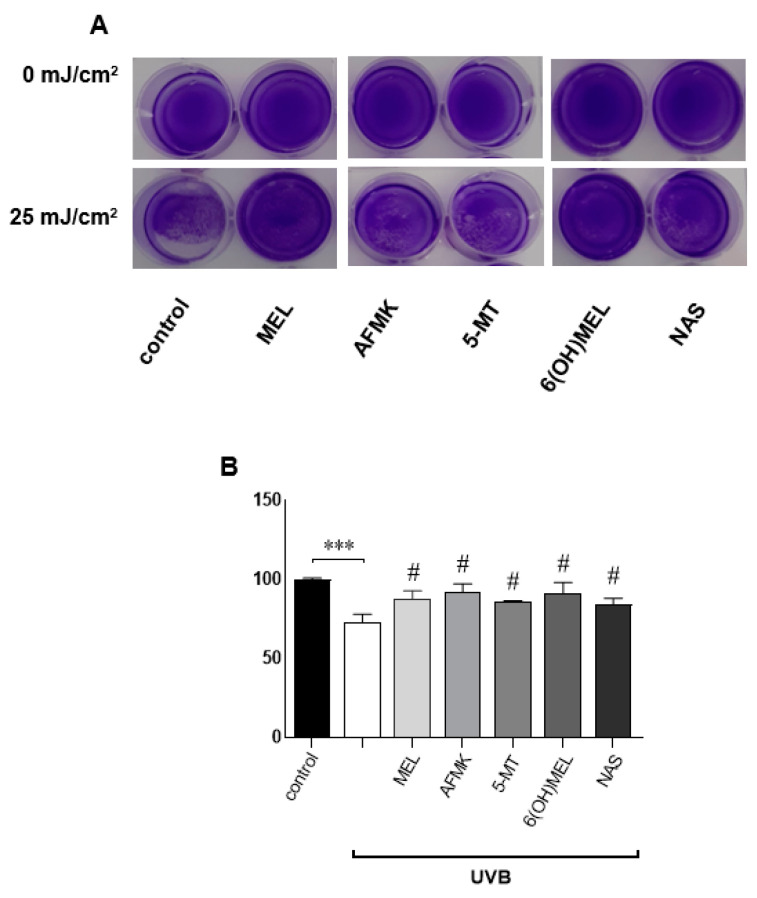
Crystal violet assessment of the protective action of metabolites of melatonin against UVB-exposed human epidermal keratinocytes. Cells were pre-incubated for 1 h with the substances (10^−3^ M), irradiated thereafter (25 mJ/cm^2^), and assessed 48 h post-UVB as described in *Materials and Methods*. Crystal violet-stained cells (**A**) were photographed and subsequently evaluated using Image J software. (**B**) Data are presented as the mean + S.E.M. of four independent experiments. Statistically significant differences are indicated as *** *p* < 0.001 versus sham-irradiated cells (control sample) and as ^#^ *p* < 0.001 for the studied compounds versus UVB-exposed cells. Values are expressed as percentages of sham-irradiated cells (0 h 0 mJ/cm^2^).

Next, we assessed the effects of MEL or its metabolites (AFMK, 5-MT, 6(OH)MEL, NAS) in terms of their attenuating action against UVB (Figure 2A–E). Pre-incubated cells with the studied substances (10^−3^–10^−9^ M) revealed a distinct protective effect ranging from 13% to 29% (10^−9^ M) and from 26% to 36% (10^−3^ M); the investigated derivatives showed similar protective efficacy. Comparatively, keratinocytes exposed to UVB (25 mJ/cm^2^) without the tested substances revealed a 47% decrease in cell viability compared to sham-irradiated cells (Figure 2, insert). Furthermore, these data were confirmed by crystal violet assays, where human keratinocytes exposed to UVB were significantly protected by the studied compounds (Figure 3A,B), with a difference ranging from 15% to 20% versus cells treated with UVB alone. 

### 3.2. Melatonin and Its Metabolites Counteract UVB-Induced Mitochondrial Disturbances

**Figure 4:**
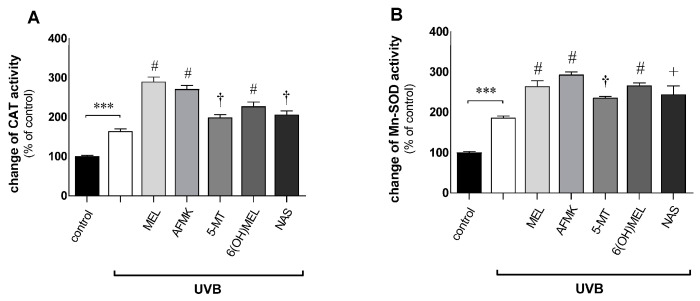
Changes in catalase (CAT) and superoxide dismutase (Mn-SOD) activity in human epidermal keratinocytes exposed to UVB. After a 1 h pre-incubation period with melatonin or its metabolites (10^−3^ M), UVB irradiation (25 mJ/cm^2^) was applied and cells were assessed 48 h post-UVB using a colorimetric assay for CAT activity (**A**) and Mn-SOD (**B**) as described in *Materials and Methods*. Data are presented as the mean +S.E.M. of six independent experiments. Statistically significant differences are indicated as *** *p* < 0.001 versus sham-irradiated cells (control sample) as ^†^ *p*< 0.05, ^+^ *p* < 0.01, ^#^ *p* < 0.001 for the studied compounds versus UVB-exposed cells. Values are expressed as percentages of sham-irradiated cells (0 h 0 mJ/cm^2^).

Epidermal keratinocytes irradiated with UVB revealed enhanced activation of catalase (CAT) and mitochondrial superoxide dismutase (Mn-SOD) related to intracellular oxidative stress (Figure 4A,B). Namely, UVB alone increased the activity of CAT and SOD by 64% and 86%, respectively. Interestingly, the presence of melatonin or its metabolites triggered responses of the anti-oxidative enzymes ranging from 35% (5-MT) to 126% (MEL) for catalase and from 49% (5-MT) to 107% (AFMK) for superoxide dismutase compared to UVB-exposed cells alone.

**Figure 5:**
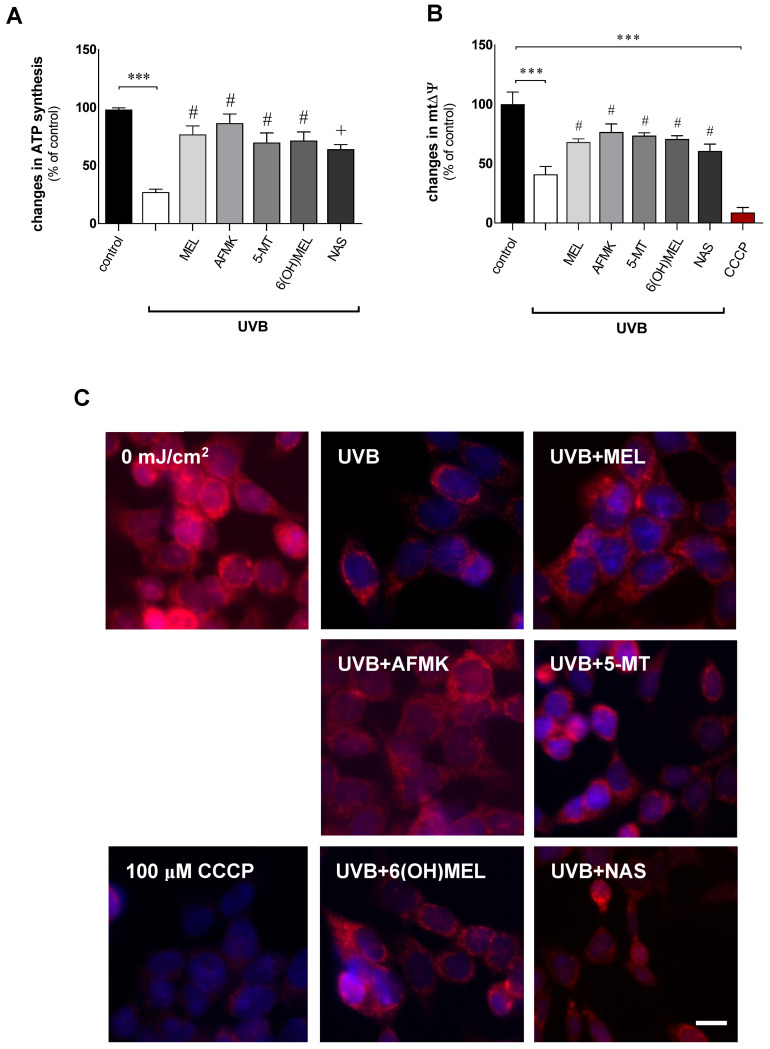
Evaluation of UVB-induced changes in ATP synthesis and the depolarization of mitochondrial transmembrane potential (mtΔΨ) in human epidermal keratinocytes. After a 1 h pre-incubation period with melatonin or its metabolites (10^−3^ M), UVB irradiation (25 mJ/cm^2^) was applied and cells were assessed 48 h post-UVB using a colorimetric assay for ATP synthesis (**A**), flow cytometry (**B**) and fluorescent microscopy (**C**) for mtΔΨ as described in *Materials and Methods*. Data are presented as the mean +S.E.M. of six independent experiments. (**A**,**B**) Statistically significant differences are indicated as *** *p* < 0.001 versus sham-irradiated cells (control sample) as ^+^ *p* < 0.01, ^#^ *p* < 0.001 for the studied compounds versus UVB-exposed cells. Values are expressed as a percentage of sham-irradiated cells (0 h 0 mJ/cm^2^). (**C**) Fluorescent images present depolarization of mtΔΨ after UVB and the protective effects of melatonin or its metabolites. Bar = 20 μm. TMRM (red) for mtΔΨ; DAPI (blue) for nuclei.

Due to oxidative stress-related changes, UVB induced deleterious alterations within the mitochondrial maintenance. We observed a 73% reduction in ATP synthesis (Figure 5A) and a 60% drop in the mitochondrial transmembrane potential (mtΔΨ) (Figure 5B) versus sham-irradiated cells. Introducing MEL or its metabolites effectively counteracted these disturbances, where the synthesis of ATP was protected by 37% (NAS), 42% (5-MT), 45% (6(OH)MEL), 49% (MEL) and by 59% (AFMK) compared to UVB-exposed cells alone. Similarly, mtΔΨ was enhanced by 20% for NAS, 33% for 5MT and by 36% for AFMK. Additionally, the dissipation of mitochondrial transmembrane potential was visualized using fluorescent imaging where the studied compounds significantly attenuated the negative effects of UVB (Figure 5C).

**Figure 6:**
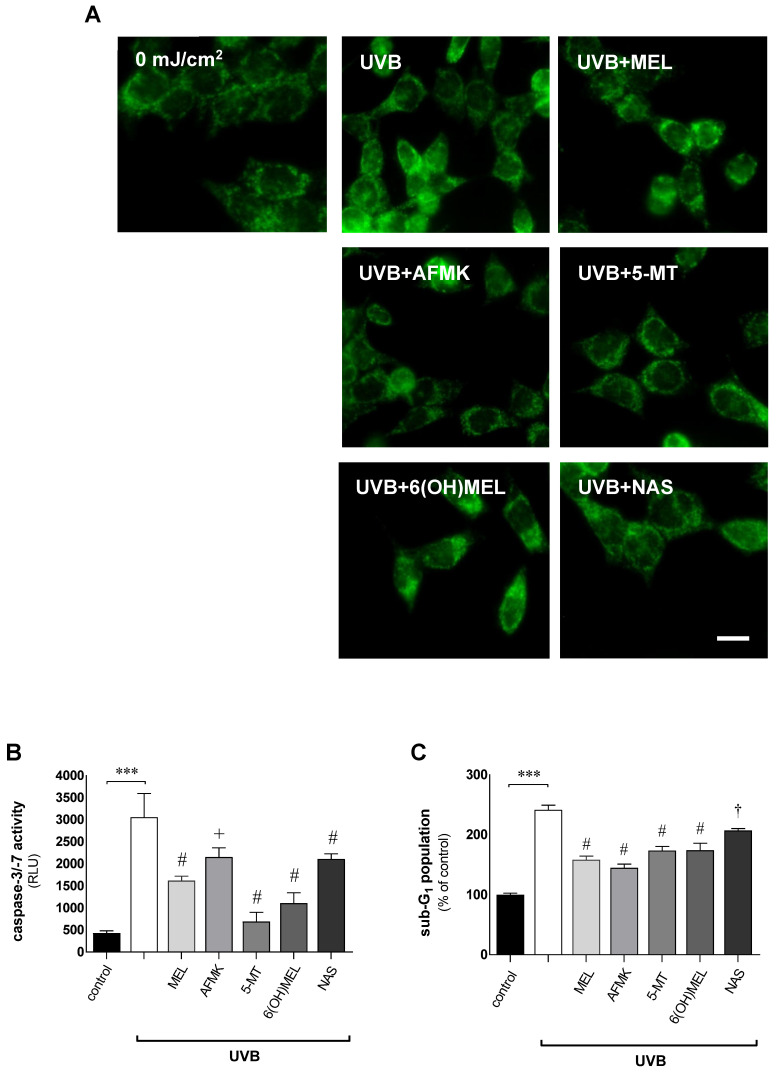
Evaluation of protective effects of MEL or its metabolites against UVB-induced cytochrome *c* release, the activation of Casp-3/-7 and the induction of sub-G_1_ population related to apoptosis in human epidermal keratinocytes. Cells were pre-incubated for 1 h, irradiated thereafter (25 mJ/cm^2^), and assessed 48 h post-UVB as described in *Materials and Methods*. (**A**) Fluorescent images present the leakage of cytochrome *c* into the cytosol after UVB irradiation and the ameliorating effect of the studied substances. Bar = 20 μm. (**B**) Assessment of Casp-3/-7 and (**C**) sub-G_1_ population was performed and data are presented as the mean + S.E.M. of four independent experiments. Statistically significant differences are indicated as *** *p* < 0.001 versus sham-irradiated cells (control sample) as ^†^ *p*< 0.05, ^+^ *p* < 0.01, ^#^ *p* < 0.001 for the studied compounds versus UVB-exposed cells. Values are expressed as percentage of sham-irradiated cells (0 h 0 mJ/cm^2^).

Since UVB decreases electron flow through the electron transport chain (ETC), oxidative phosphorylation and reduced ATP synthesis are also halted, and the downstream mitochondria-dependent apoptotic pathway is activated. Thus, UVB enhances the leakage of cytochrome *c* into the cytosol (Figure 6A), which triggers activation of the caspase cascade (Figure 6B) and the induction of apoptosis by the elevated number of sub-G_1_ cells (Figure 6C). The application of MEL or its metabolites effectively attenuates these mitochondrial dysfunctions and protects cells against UVB-induced cell death. 

## 4. Discussion

Epidermal keratinocytes forming the most outer layer of the human skin, the epidermis, are subject to the pressure of various environmental stressors [13], including UVB, which may induce elevation in ROS formation, leading to oxidative damage to many cellular components, which can create deleterious changes within intracellular compartments and organelles [53,54]. As a result, the damage to the skin leads to cell senescence, photoaging, carcinogenesis, inflammation, etc. Natural compounds, such as melatonin, have the potential to activate endogenous antioxidant defense mechanisms and enhance the protection of the skin from UVR [42]. Melatonin is a well-known free radical scavenger [55]; it detoxifies NO, H_2_O_2_ and O_2_^•−^ [38]. Due to these actions, earlier studies have proven the beneficial effects of melatonin in treatment in multiple chronic diseases; importantly, it exhibits low or an absent toxicity [56,57].

Herein, primary human epidermal keratinocytes were treated with different doses of melatonin or its derivatives. We have demonstrated that melatonin, as well as AFMK, 5-MT, 6(OH)MEL and NAS, protects cells from UVB-induced oxidative stress, in accordance with previous studies [40]. Additionally, our data showed that the exposure of keratinocytes to UVB decreased their viability, which was counteracted by melatonin and its metabolites. UVB also triggers deleterious mitochondrial alterations, i.e., the dissipation of mitochondrial transmembrane potential and a reduction in ATP synthesis, leading to the mitochondrial leakage of cytochrome *c* into the cytosol and initiating the activation of the mitochondria-dependent pathway of apoptosis via the cleavage of effector caspases (Casp-3/-7). Pre-treatment of keratinocytes with MEL or its metabolites counteracted UVB-mediated intracellular damage. The observed pattern of responses in the presence of UVB is in accordance with earlier reports carried out using keratinocytes or melanocytes [18,22,38,47,58,59,60,61]. For instance, in our studies, normal epidermal keratinocytes were pre-incubated for 1 h with the studied substances prior to UVB with doses ranging from 10^−3^ M to 10^−9^ M. Earlier reports proved that incubation with MEL at 10^−3^ or 10^−6^ M enhanced the survival of keratinocytes exposed to UVB at a dose of 30 mJ/cm^2^ [62]. The attenuating effect of the lower MEL doses was also clearly visible in our study, consistent with the expression of G protein-coupled melatonin membrane receptors type MT1 and MT2 in keratinocytes. Other reports revealed that higher doses of UVB (50 mJ/cm^2^ or 60 mJ/cm^2^) were ameliorated only with high doses of MEL, e.g., close to 10^−3^ M. This was shown in human keratinocytes and melanocytes [18,19,22,40] and human skin histocultured ex vivo [23,38,44,47]. Similarly, Janjetovic et al. [40] showed that not only MEL but also its metabolites exert prominent protective effects against UVB-induced damage in epidermal cells. The augmented survival of keratinocytes observed by us and by other authors can be secondary to the inhibition of pro-apoptotic protein and the inhibition of gene expression linked to apoptosis and oxidative stress, as reported earlier [18,19,22,23,38,40,44,47].

Melatonin, due to its highly lipophilic structure, can easily penetrate through biological membranes. It is taken up by cells, and one of the main targeted organelles for melatonin are mitochondria [36,63]. In various cell culture and animal models, melatonin exerts an astonishing diversity of biological effects when administered exogenously. Such actions include anti-inflammatory, tissue- and cell-protective, and anti-oxidative effects [4,64,65]. These effects are largely a result of the effect of melatonin on mitochondrial metabolism [36,37]. Melatonin improves the efficiency of the electron transport chain, enhances ATP generation and reduces mitochondrial ROS damage. Moreover, it promotes the activities of anti-oxidative enzymes while suppressing pro-oxidant enzymes in normal cells. The mitochondrial dynamics are in accordance with the concept that mitochondria are key players of cellular aging via excessive mitochondrial ROS production, electron transport chain defects, bioenergetic imbalance, and dysregulated mitochondrial calcium homeostasis [66,67,68,69]. Our study confirmed the concept that not only melatonin but also its derivatives effectively attenuate so-called mitochondrial dysfunction-associated senescence (MiDAS) [67,70]. Indeed, in this report, we have shown that UVB distinctly enhances CAT activity and it subsequently triggers severe alterations within the mitochondria, arresting ATP synthesis or inducing the depolarization of mitochondrial transmembrane potential. This leads to functional alterations within mitochondria by means of the release of cytochrome *c*, inducing the subsequent activation of a cascade of caspases and apoptosis. This triggers mitochondria-dependent apoptotic responses. Herein, the application of melatonin or its metabolites effectively counteracts these deleterious changes. We also note that the anti-oxidative responses regulated by melatonin have been thoroughly described and that it plays a central role in cell apoptosis or survival, involving mitochondrial cell signaling pathways via the up-regulation of sirtuins (SIRTs) [20,64,71,72,73]. Of note, we observed significant changes in CAT or the mitochondrial form of SOD (Mn–SOD). On one hand, this shows that the mitochondria are the main target for melatonin, as also confirmed for its metabolites, but these organelles are protected against UVB-induced massive oxidative stress in human keratinocytes on the other. This observation is also confirmed by the crucial role of Mn–SOD against cumulative oxidative stress events and the superior role of mitochondria-generated ROS (mROS) as the major contributor to oxidative stress in cutaneous cells.

The physiological versus pharmacological levels of melatonin have been intensively discussed [74]. Indeed, it was shown that the physiological range (10^−9^ or 10^−8^ M), similar to the pharmacological dose of 10^−3^ M, reduces UV-induced ROS formation in human leukocytes [75]. However, a stronger ROS-suppressive effect of melatonin is observed at a higher concentration, which may reflect melatonin’s direct radical scavenging properties, whereas melatonin at a lower concentration might act through the activation of membrane, cytosolic and/or nuclear receptors [9,45]. Thus, it was presented that a pharmacological dose (10^−4^ or 10^−3^ M) increases the activity of superoxide dismutase, catalase and glutathione peroxidase [76]. This is consistent with our concentration (10^−3^ M) used in later parts of our report, despite the fact that we also observed the protective effect of melatonin and its metabolites (10^−9^ M) in terms of viability assessment. Furthermore, this concentration range is in line with earlier reports where this indoleamine effectively protected keratinocytes and melanocytes from UVB-induced damage in vitro or significantly decreased levels of the DNA damage marker 8-hydroxy-2′-deoxyguanosine (8-OHdG) in an ex vivo full-thickness human skin culture [18,19,22,40,77]. Interestingly, the efficacy of high doses of externally applied melatonin or its metabolites are secondary to their endogenous production in skin cells, including melanocytes and keratinocytes, and their malignant lines [44]. Furthermore, this could explain the potent radio-protective and antioxidant role of melatonin in the skin, indicating the possibility of mitochondrial involvement. Indeed, studies have increasingly pointed to a “symbiotic” relationship between melatonin and the epidermal mitochondria, with the latter serving as the site of cutaneous melatonin biosynthesis and metabolism. It should also be added that melatonin possesses direct regulatory effects on the mitochondrial respiratory chain and antioxidant machinery. The primary metabolites of melatonin used in this study, including AFMK, 5-MT, 6(OH)MEL and NAS, are reported to accumulate in the epidermis and epidermal keratinocytes following UVB exposure [6]. Therefore, a melatonin biosynthetic machinery may also be present in the mitochondria since oocyte mitochondria can synthesize melatonin from serotonin in a cell-free system [78], as is likely the case for many other substances [36,37]. Furthermore, melatonin is metabolized in the mitochondria into active metabolites [79], which also include skin mitochondria [80]. Although it remains to be elucidated whether melatonin is synthesized in the cutaneous mitochondria, it can directly increase the electron flux through the respiratory chain and enhance ATP production by donating electrons. This was also shown for 6(OH)MEL, which promoted electron transfer from complex III to complex IV (cytochrome *c* oxidase), which may help to restore the age-dependent decline in mitochondrial bioenergetics [81,82]. Collectively, the new data uncovered in the current studies demonstrate the protective roles of MEL itself and its metabolites in the mitochondria of skin cells, indicating that these substances can be used to maintain healthy skin, to prevent different skin pathologies and as anti-aging compounds [83].

## 5. Conclusions

Evidence in the literature is clear in highlighting that UVB is one of the main factors enhancing mitochondrial disturbances related to cell senescence. Herein, we assessed the protective effects of melatonin and its metabolites against UVB in human epidermal keratinocytes. While the data indicate that mitochondria become dysfunctional in vitro, very little is known about mitochondrial functions, dynamics and structure in cells present in tissues in vivo. Therefore, we suggest that a more in-depth characterization of mitochondrial function is warranted involving other cutaneous cell types, i.e., human dermal fibroblasts or human melanocytes. Moreover, future studies should focus on the impact of melatonin on senescent markers but also on the question of what percentage of senescent cells contribute to the overall mitochondrial dysfunction observed in selected tissues during aging. Thus, there is consistency showing that mitochondria from aged tissues reveal decreased respiratory capacities and enhanced ROS generation. 

We propose that a prudent approach to the use of melatonin as an anti-aging substance could pave the way for the development of novel strategies delaying inflammation and other pathophysiologies that promote skin health.

## Data Availability

Not applicable.
