# Correction: Holtkamp et al. Ultraviolet Radiation-Induced Mitochondrial Disturbances Are Attenuated by Metabolites of Melatonin in Human Epidermal Keratinocytes. *Metabolites* 2023, *13*, 861

**DOI:** 10.3390/metabo16070458

**Published:** 2026-06-30

**Authors:** Chantal E. Holtkamp, Dawid Warmus, Klaudia Bonowicz, Maciej Gagat, Kinga Linowiecka, Agnieszka Wolnicka-Glubisz, Russel J. Reiter, Markus Böhm, Andrzej T. Slominski, Kerstin Steinbrink, Konrad Kleszczyński

**Affiliations:** 1 Department of Dermatology, University of Münster, Von-Esmarch-Str. 58, 48149 Münster, Germany; chantal.holtkamp@uni-muenster.de (C.E.H.); boehmma@ukmuenster.de (M.B.); kerstin.steinbrink@ukmuenster.de (K.S.); 2 Department of Biophysics and Cancer Biology, Faculty of Biochemistry, Biophysics and Biotechnology, Jagiellonian University, Gronostajowa 7, 30-387 Krakow, Poland; dawid.warmus@unibe.ch (D.W.); a.wolnicka-glubisz@uj.edu.pl (A.W.-G.); 3 Department of Histology and Embryology, Collegium Medicum in Bydgoszcz, Nicolaus Copernicus University in Torun, 85-092 Bydgoszcz, Poland; klaudia.bonowicz@cm.umk.pl (K.B.); mgagat@cm.umk.pl (M.G.); 4 Department of Human Biology, Faculty of Biological and Veterinary Sciences, Nicolaus Copernicus University, Lwowska 1, 87-100 Toruń, Poland; klinowiecka@umk.pl; 5 Phillip Frost Department of Dermatology & Cutaneous Surgery, University of Miami Miller School of Medicine, Miami, FL 33125, USA; 6 Department of Cell Systems and Anatomy, UT Health, Long School of Medicine, San Antonio, TX 78229, USA; reiter@uthscsa.edu; 7 Department of Dermatology, Comprehensive Cancer Center, University of Alabama at Birmingham, Birmingham, AL 35294, USA; aslominski@uabmc.edu; 8 Pathology and Laboratory Medicine Service, VA Medical Center, Birmingham, AL 35294, USA

**Error in Figure**

In the original publication [1], there was a mistake in **Figure 5**. 

The corrected **Figure 5** appears below.

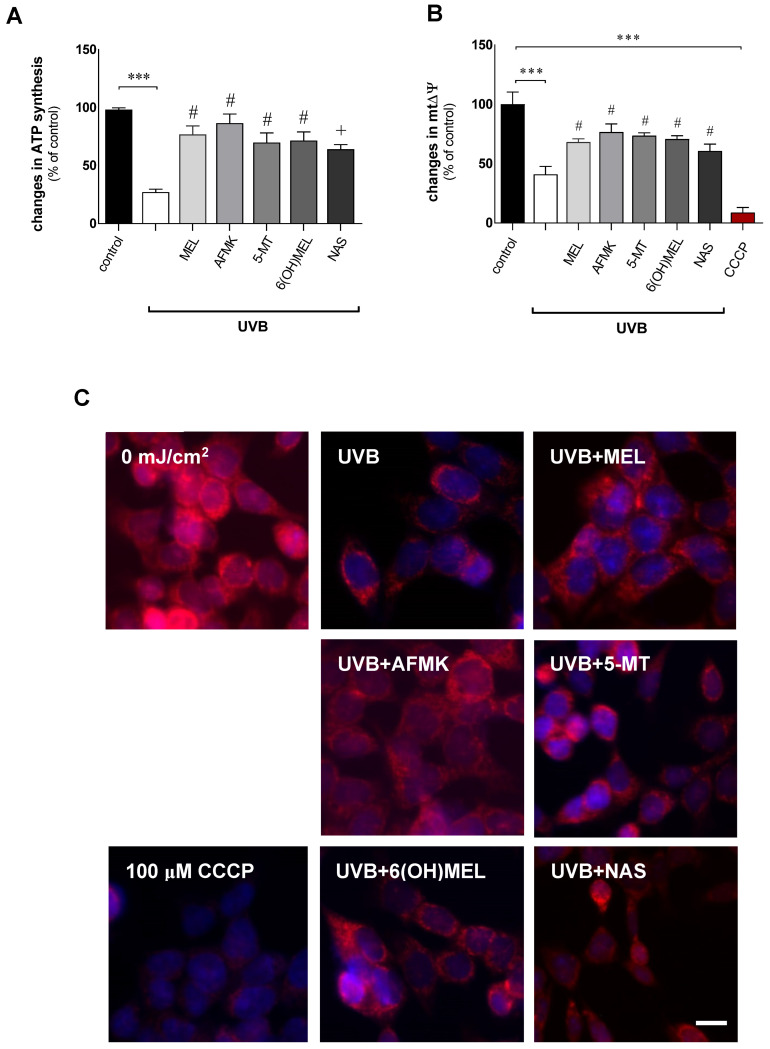



In the original publication, there was a mistake in **Figure 6**.

The corrected **Figure 6** appears below.

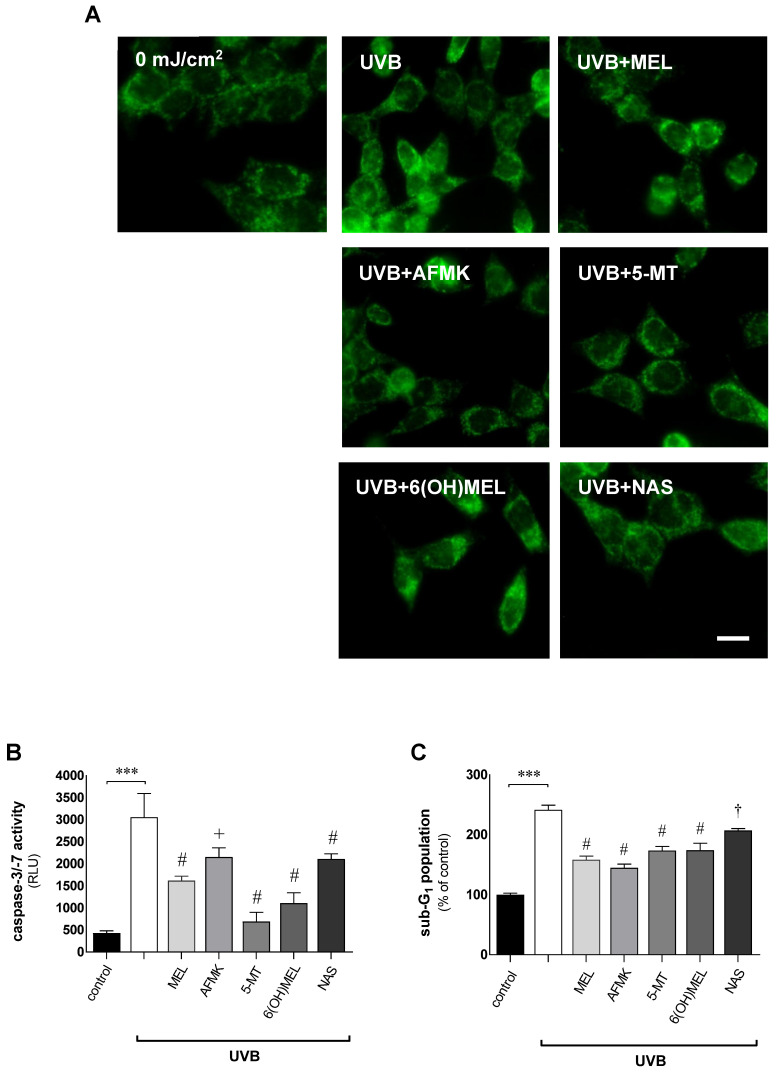



The authors state that the scientific conclusions are unaffected. This correction was approved by the Academic Editor. The original publication has also been updated.
